# 536. Utilizing BCQP to Assess the Impact of Diabetes Mellitus on Wound Healing in Burn Patients

**DOI:** 10.1093/jbcr/irag033.233

**Published:** 2026-04-06

**Authors:** Arthur Kom Sipowa, Jacob Furcolo, Tuan D Le, Melissa M McLawhorn, Bonnie C Carney, Lauren T Moffatt, Jeffrey W Shupp, Shawn Tejiram

**Affiliations:** The Burn Center at MedStar Washington Hospital Center, Washington, DC; The Burn Center at MedStar Washington Hospital Center, Washington, DC; The Burn Center at MedStar Washington Hospital Center, Grand Prairie, TX; MedStar Health, Washington, DC; The Burn Center at MedStar Washington Hospital Center, Washington, DC; The Burn Center at MedStar Washington Hospital Center, Washington, DC; MedStar Washington Hospital Center, Washington, DC; MedStar Washington Hospital Center, Washington, DC

## Abstract

**Introduction:**

Diabetes mellitus (DM) is a chronic disease that impairs tissue repair through reduced angiogenesis, neuropathy, microvascular dysfunction, and infection risk. In burn patients, these changes contribute to delayed wound healing, prolonged closure, and higher complication rates, as shown in single-institution studies. However, national-level data remain limited. This study used the Burn Care Quality Platform (BCQP) to evaluate the effect of DM on wound healing in burn patient. We hypothesized that burn patients with DM would require longer time to achieve wound closure, undergo more operative room (OR) trips, and have longer hospital length of stay (LOS) compared to non-diabetic patients.

**Methods:**

Data extracted from BCQP (2016–2018) were analyzed. Patients with DM were identified by comorbidity codes and only initial admissions with complete data were included. Patients were stratified by diabetic status and burn severity (< 20% vs ≥20% TBSA). The primary outcome was time to wound closure. Secondary outcomes included abnormal blood glucose, operative interventions, LOS, and ICU stay. Continuous variables were compared using Mann–Whitney U; categorical variables with Chi-square or Fisher’s exact test. Analyses were performed using SAS 9.4 and Prism 10.

**Results:**

Of 14 791 patients identified, 2991 had DM and 11 798 did not. Of these, 1668 patients met inclusion criteria. Median age was 51 years (IQR 35–62), 69% were male, and TBSA was 4.0% (IQR 2.0–8.8). DM prevalence was 23.2% and mortality 2.5%. Median time to 95% wound closure was 23 days (IQR 13–36) longer in DM vs non-DM (25 [14–45] vs 22 [12–34] days, *p*=.0006). Abnormal glucose was more common in DM within 24 hours (32.1% vs 5.4%, *p*<.0001) and 48 hours (24.5% vs 3.0%, *p*<.0001) after admission. DM patients underwent more OR interventions (15.8% vs 1.3%, *p*<.0001) and had longer overall LOS (9 [3–18] vs 5 [2–14] days, *p*<.0001) and ICU LOS (7 [2–15] vs 2 [1–11] days, *p*<.0001).

**Conclusions:**

DM is associated with delayed wound healing, more operative interventions, and longer LOS. Further study will be necessary to elucidate these findings.

**Applicability of Research to Practice:**

This novel multicenter analysis emphasizes the need for tailored management strategies in burn patients with diabetes to set management expectations and improve outcomes.

**Funding for the study:**

N/A.

